# Supplementary material for: Flexible Self-Powered Low-Decibel Voice Recognition Mask
Source: Sensors (Basel). 2024 May 9;24(10):3007. doi: 10.3390/s24103007 (PMC11124924; doi:10.3390/s24103007)
Supplement: Supplementary file 1 [file sensors-24-03007-s001.zip › sensors-2944960-supplementary.pdf]

sensors

Appendix A

Supporting Information

Flexible self-powered low-decibel voice recognition mask

Jianing Li[1], Yating Shi[1], Jianfeng Chen[1], Qiaoling Huang[1,2], Meidan Ye[1], Wenxi Guo[1,2,*]

[1]Department of Physics, College of Physical Science and Technology, Research Institution for Biomimetics and Soft Matter, Xiamen University, Xiamen 361005, Fujian, China
[2]Jiujiang Research Institute, Xiamen University, Jiujiang 332000, P. R. China
* Correspondence: wxguo@xmu.edu.cn;

The file includes:

Figure S1. Effect of device area and thickness on signal.
Figure S2. Testing the effect of distance on the signal.
Figure S3. Test scenarios for voice signals.
Figure S4. Confusion matrix for command-recognition results.
Figure S5. Confusion matrix for function-recognition results.
Figure S6. Confusion matrix for home-recognition results.
Figure S7. Confusion matrix for mixed recognition results.
Figure S8. Correlation coefficients between four users of the word "computer" are plotted.
Figure S9. Waveforms of other speech signals.
Table S1. Progress of TENG in Speech Recognition Applications.
Table S2. The comparison of current speech recognition technologies.
Table S3. Experimental instruments and equipment.

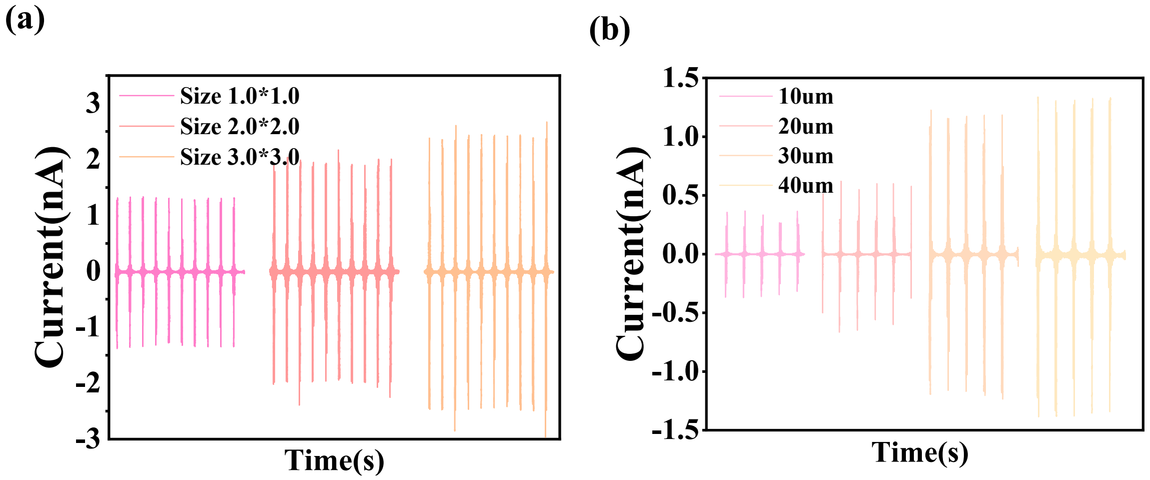

Figure S1. Effect of device area and thickness on signal.

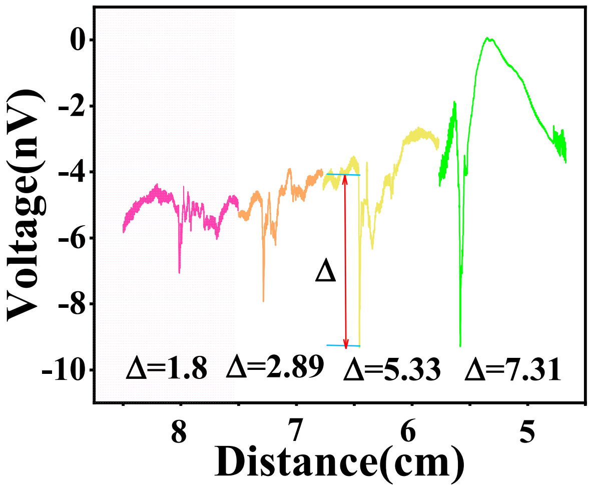

Figure S2. Testing the effect of distance on the signal.

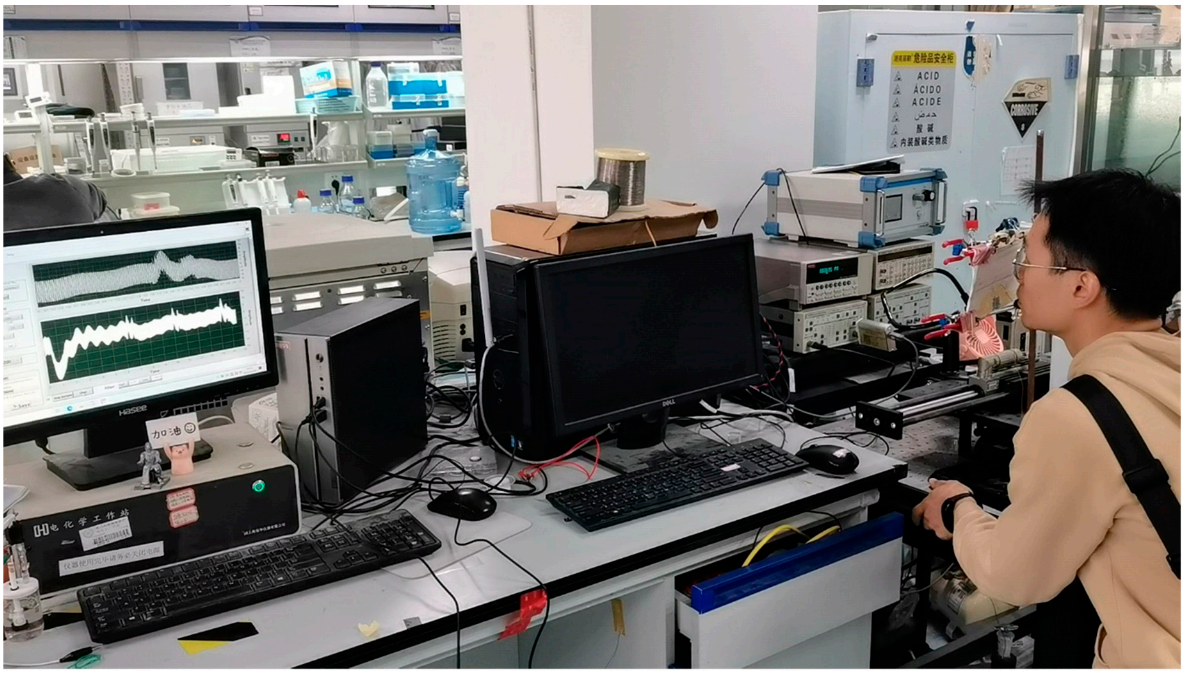

Figure S3. Test scenarios for voice signals.

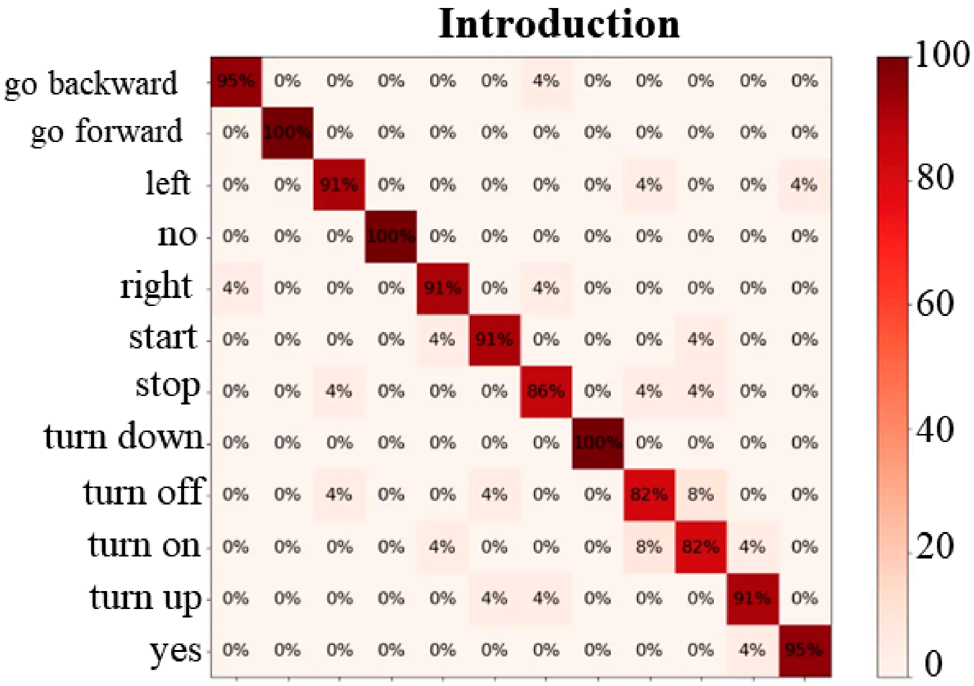

Figure S4. Confusion matrix for command- recognition results.

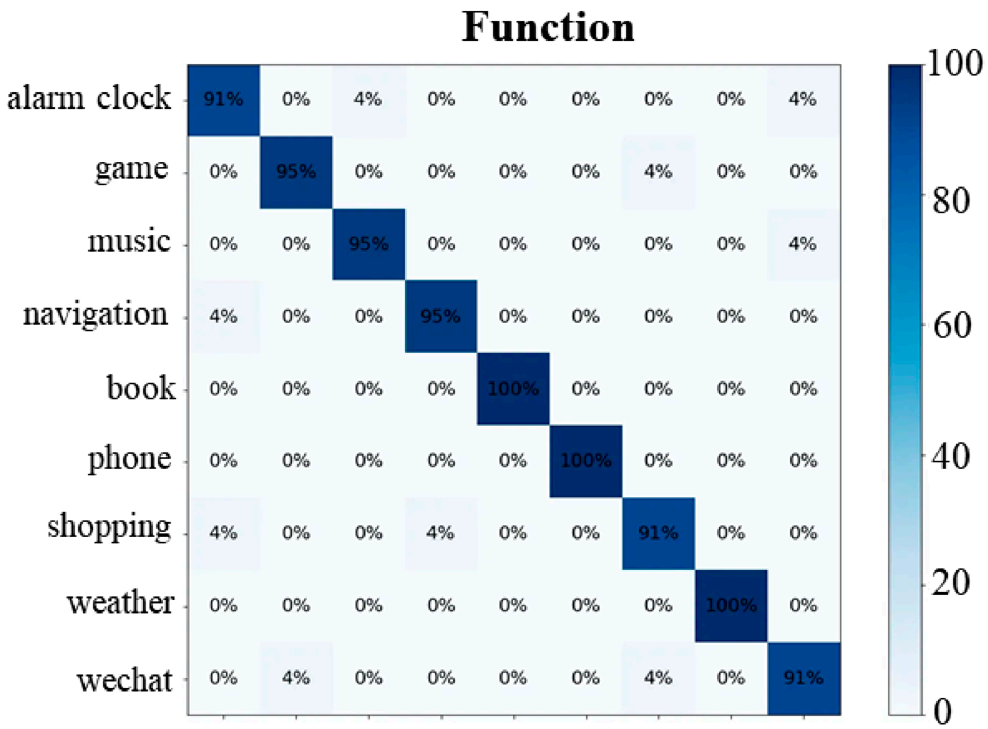

Figure S5. Confusion matrix for function- recognition results.

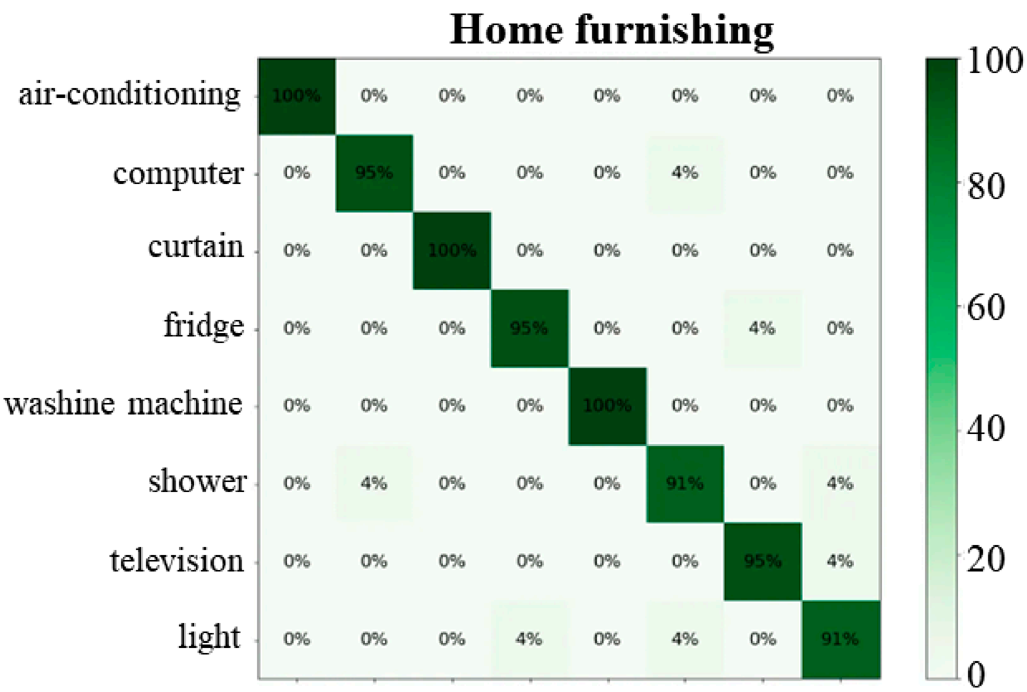

Figure S6. Confusion matrix for home-recognition results.

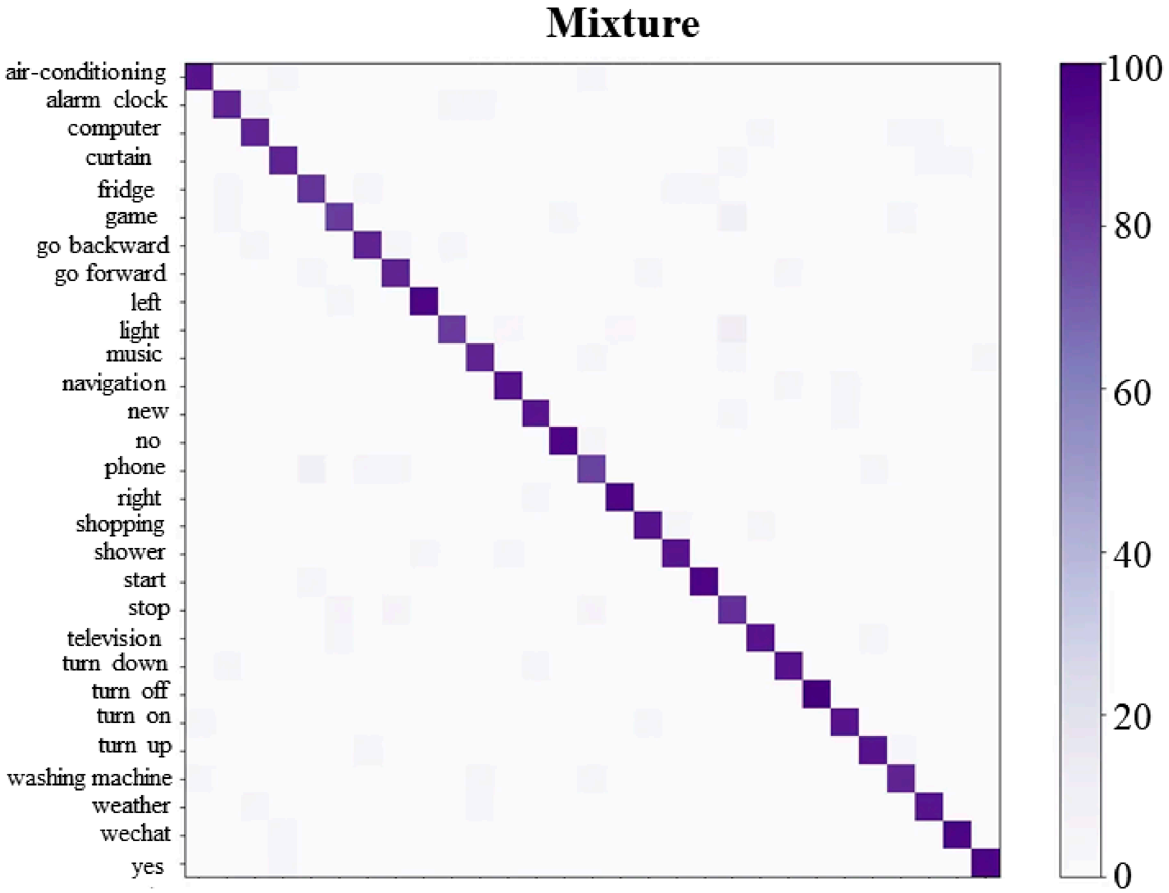

Figure S7. Confusion matrix for mixed recognition results.

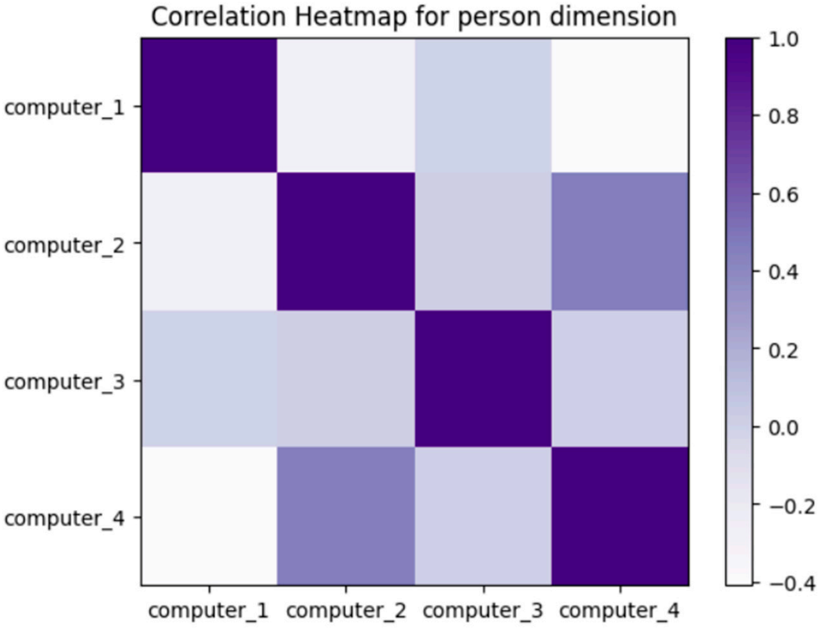

Figure S8. Correlation coefficients between four users of the word "computer" are plotted.

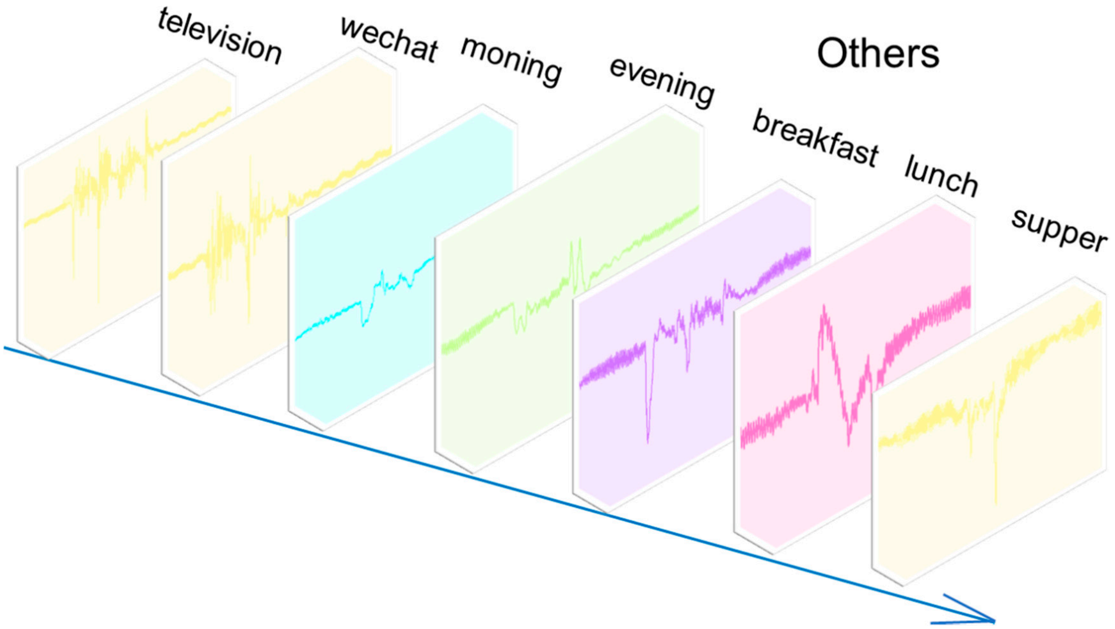

Figure S9. Waveforms of other speech signals.

Table S1. Progress of TENG in Speech Recognition Applications.

**References**	**Materials**	**Signal source**	**Noise resistance**	**Accuracy**	**Application scenarios**	**Silent / Voiced**
**[1]**	LIG/PI	Sensing throat vibrations.	——	97.9%(Three words）	Detecting different words and sentences	Voiced
**[2]**	TPU/Ionic electrode	Sound waves causing sensor vibration.	~87.3 db	——	Biometric recognition as a noise dosimeter	Voiced
**[3]**	NFCF/PE/Ag	Sensing throat vibrations.	~91db	92.3%（Seventeen words）	Semantic detection for the deaf and mute	Voiced
**[4]**	LIG/PET/PI	Thermal acoustic effect	70——115db	96.63%（Thirty vocabulary words）	Identifying the content of the sound, the speaker's emotions, and identity.	Voiced

**[5]**	Nylon/PVC/PI/Sponge/Copper	Perceiving lip movements	——	94.5%（Twenty vocabulary words）	Silent communication through lip reading	Voiceless
**[6]**	Ag/FEP/Fibroin/Cu/Acrylic	Sound waves causing sensor vibration.	~95db	97%(Voiceprint recognition for four individuals）	Speech recognition and voiceprint recognition.	Voiced
**[7]**	PDMS/PEDOT/Acetone/IPA	Dual-mode Perception of Facial Muscle Movement and Speech Recognition	——	Classification accuracy for speech/facial expressions is 93.3%.	Recognizing semantics and facial expressions.	Voiced
**[8]**	PTFE/ITO/Nylon/PET	Sensing throat vibrations	——	The Equal Error Rate (EER) is below 1.6%.	Perceiving speech signals through the throat and conducting biometric authentication.	Voiced
**[9]**	Metal mesh/Plastic ring/Metal ring/Metal Plate/Circuit board	Sound waves causing sensor vibration.	~100 db	——	Music recording and wireless communication	Voiced

Table S2. The comparison of current speech recognition technologies.

Comparison aspect	Traditional speech recognition	Silent speech recognition	References
**Signal source**	Audio signal	Electromyography (EMG) or other biological signals	[10][11]
**Noise resistance**	Generally below 100 decibels	Not necessarily	[12][13]
**Accuracy**	High in controlled environments	Generally lower but can be increased	[14]
**Portability**	High (requires only one microphone)	Moderate (requires specialized sensors)	[15]
**Privacy**	Relatively low (can be overheard)	Relatively high (better confidentiality)	[12][16]
**Innovation point**	Voice activity detection, multilingual support, and integration with multiple devices	Neural decoding, sensor enhancements, and silent processing mechanisms	[17]
**Application domain**	Common consumer use, most smart devices	Special environments (such as high-noise industries, secure communication)	[10][12][18]

Based on the research and analysis above, it is evident that the current technology of silent speech recognition needs improvement in terms of portability, accuracy, etc. Additionally, it should possess universality, enabling it to be applied in most smart devices, similar to conventional speech recognition.

Table S3. Experimental instruments and equipment.

Experimental Instrument	Instrument Model	Manufacturer
**Micro Force Tensile Testing Machine**	JHY-5000	Xiamen Jinheyuan Technology Co., Ltd., China
**Electrometer**	Keithley 6514	Keithley Instruments, Inc., Cleveland, Ohio, USA
**Current Amplifier**	SR570	Stanford Research Systems, Sunnyvale, CA, USA
**Signal Generator**	CD-XG30P	Jiangsu Lianeng Electronic Technology Co., Ltd,China
**Enzyme-linked Immunosorbent Assay (ELISA) Reader**	SpectraMax M2	Meigu Molecular Instruments Co., Ltd,China
**Fluorescence Microscope**	LEICA DMi8	Jingyi Xingye Technology Co., Ltd,China

References

1. Xia, S.-Y.; Long, Y.; Huang, Z.; Zi, Y.; Tao, L.-Q.; Li, C.-H.; Sun, H.; Li, J. Laser-Induced Graphene (Lig)-Based Pressure Sensor and Triboelectric Nanogenerator Towards High-Performance Self-Powered Measurement-Control Combined System. Nano Energy 2022, 96, 107099.

2. Wang, H.L.; Guo, Z.H.; Pu, X.; Wang, Z.L. Ultralight Iontronic Triboelectric Mechanoreceptor with High Specific Outputs for Epidermal Electronics. Nano-Micro Lett. 2022, 14, 86.

3. Zhao, J.; Chen, D.; Zhao, L.; Shi, Y.; Guo, S.; Zhu, Z.; Liu, J.; Li, W.; Lei, W.; Chen, H. Self-powered speech recognition system for deaf users. Cell Rep. Phy. Sci. 2022, 3, 101168.

4. Sun, H.; Gao, X.; Guo, L.Y.; Tao, L.Q.; Guo, Z.H.; Shao, Y.; Cui, T.; Yang, Y.; Pu, X.; Ren, T.L. Graphene-based dual-function acoustic transducers for machine learning-assisted human-robot interfaces. Infomat 2023, 5, 66-79.

5. Lu, Y.; Tian, H.; Cheng, J.; Zhu, F.; Liu, B.; Wei, S.; Ji, L.; Wang, Z.L. Decoding lip language using triboelectric sensors with deep learning. Nat. Commun. 2022, 13, 1401, doi:10.1038/s41467-022-29083-0.

6. Luo, H.; Du, J.; Yang, P.; Shi, Y.; Liu, Z.; Yang, D.; Zheng, L.; Chen, X.; Wang, Z.L. Human-Machine Interaction via Dual Modes of Voice and Gesture Enabled by Triboelectric Nanogenerator and Machine Learning. ACS Appl. Mater. Interfaces 2023, 15, 17009-17018, doi:10.1021/acsami.3c00566.

7. Lee, J.P.; Jang, H.; Jang, Y.; Song, H.; Lee, S.; Lee, P.S.; Kim, J. Encoding of multi-modal emotional information via personalized skin-integrated wireless facial interface. Nat. Commun. 2024, 15, 530, doi:10.1038/s41467-023-44673-2.

8. Yang, J.; Chen, J.; Su, Y.; Jing, Q.; Li, Z.; Yi, F.; Wen, X.; Wang, Z.; Wang, Z.L. Eardrum-inspired active sensors for self-powered cardiovascular system characterization and throat-attached anti-interference voice recognition. Adv. Mater. 2015, 27, 1316-1326, doi:10.1002/adma.201404794.

9. Yang, H.; Li, Q.; Zhang, X.; Li, X.; Yang, Q.; Hu, Y.; Xi, Y.; Wang, Z.L. High-sensitive and ultra-wide spectrum multifunctional triboelectric acoustic sensor for broad scenario applications. Nano Energy 2022, 104, 107932, doi:10.1016/j.nanoen.2022.107932.

10. Huang, X.; Acero, A.; Hon, H.-W.; Reddy, R. Spoken language processing: A guide to theory, algorithm, and system development; Prentice Hall: Upper Saddle River, N.J., 2001.

11. Bilmes, J.A. Graphical models and automatic speech recognition. In Proceedings of the Mathematical Foundations of Speech and Language Processing, New York, NY, 2004, 2004; pp. 191-245.

12. Denby, B.; Schultz, T.; Honda, K.; Hueber, T.; Gilbert, J.M.; Brumberg, J.S. Silent speech interfaces. Speech Commun. 2010, 52, 270-287.

13. Herff, C.; Schultz, T. Automatic speech recognition from neural signals: a focused review. Front. Neurosci. 2016, 10, 204946.

14. Maier-Hein, L.; Metze, F.; Schultz, T.; Waibel, A. Session independent non-audible speech recognition using surface electromyography. In Proceedings of the IEEE Workshop on Automatic Speech Recognition and Understanding, Cancun, Mexico, 27 Nov.-1 Dec. 2005, 2005; pp. 331-336.

15. Meltzner, G.S.; Sroka, J.J.; Heaton, J.T.; Gilmore, L.D.; Colby, G.; Roy, S.H.; Chen, N.; De Luca, C.J. Speech recognition for vocalized and subvocal modes of production using surface EMG signals from the neck and face. In Proceedings of the INTERSPEECH, Brisbane, Australia, September 22-26, 2008; pp. 2667-2670.

16. Sun, H.; Zhang, X.; Zhao, Y.; Zhang, Y.; Zhong, X.; Fan, Z. A novel feature optimization for wearable human-computer interfaces using surface electromyography sensors. Sensors 2018, 18, 869.

17. Jorgensen, C.; Binsted, K. Web Browser Control Using EMG Based Sub Vocal Speech Recognition. In Proceedings of the Proceedings of the 38th Annual Hawaii International Conference on System Sciences, Big Island, HI, USA, 6-6 Jan. 2005; pp. 294c-294c.

18. Xiong, X. Fundamentals of speech recognition. Available online: https://slpcourse.github.io/materials/lecture_notes/Xiao_Guest_lecture_ASR.pdf (accessed on 21 April 2024).
